# Toxicity Evaluation of the Naphthalen-2-yl 3,5-Dinitrobenzoate: A Drug Candidate for Alzheimer Disease

**DOI:** 10.3389/fphar.2021.607026

**Published:** 2021-05-10

**Authors:** Fareeha Anwar, Uzma Saleem, Atta-Ur Rehman, Bashir Ahmad, Matheus Froeyen, Muhammad Usman Mirza, Lee Yean Kee, Iskandar Abdullah, Sarfraz Ahmad

**Affiliations:** ^1^ Riphah Institute of Pharmaceutical Sciences, Riphah International University, Lahore, Pakistan; ^2^ Riphah Faculty of Pharmaceutical Sciences, Riphah International University, Islamabad, Pakistan; ^3^ Department of Pharmacology, Faculty of Pharmaceutical Sciences, Govt. College University, Faisalabad, Pakistan; ^4^ Department of Pharmacy, Forman Christian University, Lahore, Pakistan; ^5^ Department of Pharmaceutical and Pharmacological Sciences, Rega Institute for Medical Research, Medicinal Chemistry, University of Leuven, Leuven, Belgium; ^6^ Department of Chemistry, Faculty of Science, Universiti Malaya, Kuala Lumpur, Malaysia

**Keywords:** acute oral toxicity, subacute toxicity, teratogenicity, oxidative stress markers, biochemical parameters

## Abstract

The presented study was designed to probe the toxicity potential of newly identified compound naphthalen-2-yl 3,5-dinitrobenzoate (SF1). Acute, subacute toxicity and teratogenicity studies were performed as per Organization of economic cooperation and development (OECD) 425, 407, and 414 test guidelines, respectively. An oral dose of 2000 mg/kg to rats for acute toxicity. Furthermore, 5, 10, 20, and 40 mg/kg doses were administered once daily for 28 days in subacute toxicity study. Teratogenicity study was performed with 40 mg/kg due to its excellent anti-Alzheimer results at this dose. SF1 induced a significant rise in Alkaline Phosphatases (ALP), bilirubin, white blood cells (WBC), and lymphocyte levels with a decrease in platelet count. Furthermore, the reduction in urea, uric acid, and aspartate transaminase (AST) levels and an increase in total protein levels were measured in subacute toxicity. SF1 increased spermatogenesis at 5 and 10 mg/kg doses. Teratogenicity study depicted no resorptions, early abortions, cleft palate, spina bifida and any skeletal abnormalities in the fetuses. Oxidative stress markers (Superoxide dismutase (SOD), Catalase (CAT), and glutathione (GSH) were increased in all the experiments, whereas the effect on melanoaldehyde Malondialdehyde (MDA) levels was variable. Histopathology further corroborated these results with no change in the architectures of selected organs. Consequently, a 2000 mg/kg dose of SF1 tends to induce minor liver dysfunction along with immunomodulation, and it is well below its *LD*_*50*_. Moreover, it can be safely used in pregnancy owing to its no detectable teratogenicity.

## Highlights:

● *LD*_50_ of SF1 is greater than 2000 mg/kg.
● No teratogenic potential was assessed in animals treated with SF1.
● SF1 showed immunostimulatory action by increasing the WBC levels.
● Repeated administration of SF1 showed a change in biochemical markers at 40 mg/kg dose level.

## Introduction

Toxicity profiling is cardinal in the screening and development of new drugs before clinical trials (Arome and Chinedu, 2013). The key parameter behind the toxicity studies of the compounds is to ensure the protection of the exposed population against those substances (Morton, 1998). International regulatory bodies for the toxicity studies (OECD, GSH, EPA, EEC, etc.) determine the potential hazards and risks of the substances on the human beings by comparing the dose-related toxic effects that occur in the animal models (Lewis et al., 2002). Toxicity studies using animal models provide several advantages including controlled duration of exposure, the easy examination of all tissues and estimation of various biochemical and pathological markers (Arome and Chinedu, 2013; Dandekar and et al., 2010). The benefits of the toxicity profiling not only lie in the identification of possible hazards, but it also characterizes potentially toxic effects that can be produced by the test compounds on different doses and on different treatment time durations. The importance of toxicity studies emerged in the 1960s when thousands of children were born with severe congenital disabilities due to the use of anticancer drug thalidomide; commonly known as thalidomide catastrophe (Klaassen, 1996).

Toxicological profiling of the new drugs provides a relationship between a therapeutic dose and a toxic dose. After extensive toxicity studies, researchers can decide whether the drug could be subjected to clinical trials or not (Aneela et al., 2011). The results of toxicity studies in animals determine the safety of the new drugs. This safety encourages the suitability of the drug toward its therapeutic application (Jothy et al., 2011). According to the OECD guidelines, rats are recommended for the toxicity study models (OCED Guideline for Testing of Chemicals No, 1995; OCED, 2002).

Naphthalene, a bicyclic aromatic compound provides a broader platform for the synthesis of new compounds of pharmaceutical interest. Cost-effective and easy nucleophilic substitutes make it more important for the scientist in the discovery of new drugs. Naphthalene nucleus emerges as the building block in the discovery of new therapeutic drugs with lesser side effects. Many drugs are available in the market containing naphthalene nucleus and cover a broader range of diseases like antimicrobials, antihypertensive, antimalarials and antitubulin. Several naphthalene based drugs such as nafcillin, terbinafine and naftifine are armamentariums of the therapeutics being used presently (Desai et al., 2012).

**FIGURE 1:**
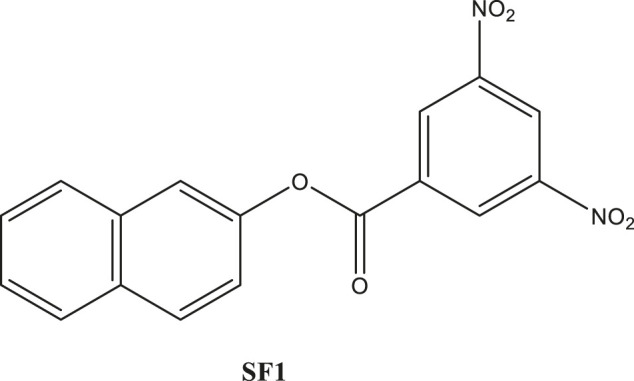
Chemical structure of naphthalen-2-yl 3,5-dinitrobenzoate (SF1).

In the present era, the *in silico* studies have greatly facilitated the selection of promising compounds for bench investigations. Target-oriented syntheses of the small molecules are planned effectively with retrosynthetic analysis. Target-oriented syntheses of the small molecules have the ability to modulate the disease-related biological pathways through the binding with targeted therapeutic protein (Harvey, 2008; Hughes et al., 2011). We analyzed different compounds through extensive *in silico* analysis and identified naphthalen-2-yl 3,5-dinitrobenzoate (SF1), a naphthalene derivative, with promising binding with acetylcholinesterase enzyme (AChE). It was synthesized and evaluated for its efficacies toward *in vitro* and *in vivo* AChE inhibition (Iman et al., 2018). The synthesis, characterization, *in silico* and *in vitro* examinations of SF1 were reported in our previous study (Arome and Chinedu, 2013). The present study aimed to evaluate acute toxicity, subacute toxicity, and teratogenicity profile of SF1 in rat model (Figure 1).

## Materials and Methods

### Drugs and Chemicals

Pyrogallol solution and alcian blue were purchased from Oxford Labs (India). Ellman’s reagent (DTNB) and alizarin red S were purchased from Omicron Sciences Limited (United Kingdom). Follin’s reagent, carboxymethyl cellulose, picric acid, Ethylenediamine tertracetic acid (EDTA), N-1-naphthylethyleneamine dihydrochloride, sulfanilamide, phosphoric acid, thiobarbituric acid, sodium phosphate dibasic heptahydrate and sodium phosphate monobasic monohydrate, sodium carbonate, sodium hydroxide, copper sulfate, and sodium-potassium tartrate, and Griess reagent were purchased from Sigma Aldrich United States. Interleukin 6 (IL-6) (Cat#EH21L6), histamine (KA2589), testosterone (MA5-14715), and nuclear factor kappa light chain enhance of activated B cells (NF-κB) (Cat#85–86081-11) were purchased from Thermo Fischer Scientific (United States).

### Experimental Animals

Adult wistar rats, 2–3 months old, weighing 200–300 g were housed in the animal house of Riphah Institute of Pharmaceutical Sciences, Riphah International University, Lahore Campus, Pakistan. The animals were provided with the standard environmental conditions 22 ± 2°C temperature, 40–50% humidity, 12:12 h light/dark cycle, and free access to food and water. Acute oral toxicity, subacute oral toxicity and teratogenicity experimental protocols were approved from Ethical Committee of Riphah Institute of Pharmaceutical Sciences (Research Ethical Committee REC), Lahore campus, with the voucher number of REC/RIPS-LHR/035 for further considerations under the rules and regulations of National Institute of Health (NIH, United States) guide for the care and use of laboratory animals.

### Acute Toxicity

Acute oral toxicity was performed according to the OECD 425 guidelines (2001) (Organization for Economic Corporation and Development). Healthy adult Wistar rats (*n* = 5) were fasted overnight but had free access to water. Initially, only one animal received SF1 (2000 mg/kg) via the oral route and was observed for 24 h. If there was no mortality, another group of four animals was orally administered with a single dose of SF1 (2000 mg/kg). Animals were observed for general behavior, any change in weight, allergic condition and mortality for 14 days (Choi et al., 2019).

### Subacute Toxicity

The experimental protocol for subacute toxicity study was performed according to the OECD 407 guidelines (2008). Male and female healthy Wistar rats were used in the study. Each group consisted of 10 animals in which five were male, and five were female. Animals were divided into five groups (*n* = 50). Group I received carboxymethylcellulose (1% CMC) (1 ml/kg). Group II-IV was administered 5, 10, 20, and 40 mg/kg dose of SF1 for consecutive 28 days via oral route once daily. Any change in the weight and any sign of physical abnormalities were noted on each day of treatment (Yamasaki et al., 2002; Gelbke et al., 2007).

### Teratogenicity

The experimental protocol was designed according to the OECD guidelines 414 (2001) for teratogenicity. Female rats were used in this study with three females and one male housed in a cage for mating. The day at which the vaginal plug was observed, labeled as start day of gestation. Pregnant females were divided into two groups (*n* = 20). Group I served as a control group and received CMC (1 ml/kg), and group II received SF1 (40 mg/kg). Both the groups received their respective treatments from gestation 5th to 15th day via the oral route. C-section was performed on the 19th day of the gestation period (Saeed et al., 2020). Fetuses were carefully removed and weighed separately. The number of fetuses and crown-rump length were noted, and deformities, including growth retardation, hematoma, and abnormalities in skull and bones were observed (Buschmann, 2013; Saeed et al., 2020).

#### Staining Technique of Fetal Skeleton

Fetuses were double-stained for analyzing any skeletal abnormalities caused by the drug during pregnancy (Menegola et al., 2001). For this purpose, fetuses of each group (control and treated) were placed overnight in the 4% of saline solution at 4°C. The skin was macerated and removed carefully, along with muscles and organs. The skeleton of the fetuses was then kept into the acidic solution (alzarian red) at pH 2.8 for 24 h, and then moisture from the sample was removed by placing the sample in absolute alcohol. The dehydrated skeletal specimens were kept in the basic solution (alcian blue) for at least 24 h, and the solution was refreshed twice during this time. This step helped in the removal of adipose tissues from the skeleton. The stained samples were placed in the clearing solution for at least 6–8 h (Ehlers et al., 1996; Menegola et al., 2001). The skeletons were preserved in 1:1 ethanol (70%) and glycerine solution. Stained skeletons were analyzed under a dissecting microscope for the observation of bone ossification, spina bifida, cleft palate, ribs deformities and limb deformities.

#### Wilson’s Technique for Soft Tissue Examination

Wilson’s technique was used for the analysis of soft tissue defects during teratogenicity studies. It gives a quick and rapid gross examination of tissues. Fetuses from each group were soaked in the bouin solution (saturated solution of Picric acid) for 8–10 days. After fixation, fetuses were soaked into distilled water for the removal of any irritant fume of picric acid (Barrow and Taylor, 1969). Vertical cuts were made for the head and body to observe malformations. Selected organs were removed carefully and examined for hydrocephalus, cardiomegaly, hydronephrosis, etc.

### Sample Collection for Sperm Analysis

The collection of sperms was made by using the diffusion method (Cartner et al., 2007). After cervical dislocation orchidectomy was performed by castration method. Testicles were oozed out by making the incision on the pre-scrotal region (Saba et al., 2009). Cauda epididymis was isolated and poured into the Petri dish containing phosphate buffer (pH 7.4). Spermatozoa were diffused out in the buffer solution by swirling the Petri plate at 37°C. Sperm suspension was picked out and analyzed for sperm count and morphological parameters (normal, hookless, bent tail, coiled tail, tailless and fused).

### Hematological and Biochemical Analysis

At the end of each study, animals were given anesthesia by using the 3–5% isoflurane with oxygen. Blood samples were collected for the hematological and biochemical studies through cardiac puncture. The hematological analysis was carried out by using a hematology analyzer from Novamed, United States. The hematological parameters evaluated included: WBC, RBC, platelet count, hemoglobin, mean corpuscular hemoglobin (MCH), Hematocrit, mean corpuscular volume (MCV), mean corpuscular hemoglobin concentration (MCHC), and differential leukocyte numbers including granulocytes, lymphocytes, and monocytes. For the biochemical analysis, whole blood and blood with anticoagulant (3.8% sodium citrate) were centrifuged at 1,400×*g* for 10–15 min. Serum and plasma were separated for the estimation of different biochemical parameters including AST, Alanine transaminase (ALT), ALP, bilirubin, total protein, uric acid, creatinine, urea, cholesterol, triglycerides (TG), low density lipoprotein (LDL), high density lipoprotein (HDL), testosterone, TSH (thyroid stimulating hormone), T_3_ and T_4_ (Konan et al., 2007; Steinberg et al., 2019). All the biochemical parameters were estimated by using their specific kits.

### Oxidative Stress Markers

After blood collection, an autopsy was performed for the observation of major organs, including heart, kidney, brain, liver, stomach, and spleen in each study. The organs were carefully removed and weighed. Tissue homogenates of each tissue were prepared in phosphate buffer (0.1 M, pH 7.4) using 1 g tissue to 10 ml buffer ratio. Tissue homogenates were centrifuged at 6,000 rpm at 4°C for 10 min. The supernatant was collected and used for the estimation of oxidative stress markers like SOD, CAT, GSH, and MDA. All the experiments were performed in a triplicate manner (Hira et al., 2019).

#### Superoxide Dismutase

0.1 ml of tissue homogenate was mixed with 2.8 ml, and 0.1 ml potassium phosphate buffer (pH 7.4, 0.1 M) and pyrogallol solution were added. Absorbance was noted at 325 nm (Saleem et al., 2019). Values of SOD were calculated by using the regression line of the standard SOD curve.

Y=0.0095X+0.1939,

#### Catalase

Tissue homogenate (0.05 ml) was mixed with 1.95 ml of phosphate buffer (50 mM, pH 7) and 1 ml of hydrogen peroxide (30 mM) solution. The mixture was analyzed using a spectrophotometer at 240 nm wavelength (Mir et al., 2019). CAT Levels in each tissue was calculated by using the following formula:

$$\text{CAT}\;\text{activity}=\frac{\text{OD}}{E}\times \text{vol}.\text{of}\;\text{sample}\times \text{mg}\;\text{of}\;\text{protein},$$

OD = Change in absorbance per minute.E = Extinction coefficient of hydrogen peroxide (0.071 mmol/cm).Protein contents were estimated using the Lowry’s method (Hira et al., 2020).

#### Glutathione

Trichloroacetic acid (1 ml, 10%), tissue homogenate (1 ml), phosphate buffer (4 ml, pH 8) and DTNB (0.5 ml) were mixed and absorbance measured at 412 nm (Saeed et al., 2020). The formula for the estimation of GSH level is as under:

$$\text{GSH}=Y-\frac{0.00314}{0.034}\times \frac{\text{DF}}{\text{BT}}\times \text{VU},$$

DF = dilution factor, BT = tissue homogenate, VU = volume used, Y = absorbance at 412 nm.

#### Malondialdehyde

Thiobarbituric acid (TBA) reagent (3 ml) was mixed with 1 ml of tissue homogenate. Shake well and incubate the mixture for 15 min at room temperature. After incubation the mixture was cooled on an ice bath and centrifuged. The supernatant was taken out and used for the measurement of MDA at 532 nm (Saeed et al., 2020). Following formula was used for the estimation of MDA levels in tissues:

$$\text{Conc}.\text{of}\;\text{MDA}=\frac{Abs\;532\times 100\times V_{t}}{1.56\times 105\times W_{t}\times V_{u}},$$

where *V*_*t*_ = total volume of mixture, *W*_*t*_ = weight of dissected tissue, *V*_*u*_ = aliquot volume.

### Protein Analysis Using ELISA Method

Blood serum was used for the ELISA analysis. Histamine, testosterone, IL-6, and NF-κB were estimated by using their specific ELISA kits. Each protein was combined with HRP to make an antigen-antibody-enzyme-antibody complex. The complex was mixed with TBM solution, and the reaction was stopped by adding the stop solution. The reaction mixture was observed spectrophotometrically at 450 nm. The concentration of each protein in the tissue homogenate was calculated by using their specific standard curve (DeMattos et al., 2002).

### Histopathological Studies

Excised tissues (heart, brain, liver, kidney, pancreas, testis, ovaries, and stomach) were fixed in 4% paraformaldehyde, embedded in paraffin, and sliced in 5 µm sections. The sliced sections were fixed onto the slides and stained with hematoxylin and eosin stain and examined under the microscope for histopathological changes in each study (Thenmozhi et al., 2015).

### Statistical Analysis

The data was expressed as mean ± SEM. Graphpad prism (v. 5.0) was used for the interpretation of experimental data. One-way ANOVA or two ways ANOVA were used for the statistical analysis of the data followed by Tukey comparison and Bonferroni post hoc test. *p* < 0.05 was considered as a level of significance. *p* < 0.01 and *p* < 0.001 labeled as moderate and highly significant levels.

## Results

### Behavior and Mortality in Acute Toxicity

A high single dose of SF1 (2000 mg/kg) did not induce any mortality, and no visible sign and symptoms of toxicity were observed during 14 days of observation. The results indicate that *LD*_*50*_ of SF1 is at a dose considerably higher than 2000 mg/kg. To understand behavioral changes, various observations were made during this study, and the results are tabulated in Table 1.

**TABLE 1:** Behavioral changes after treatment with a single dose of SF1 (2000 mg/kg) in acute oral toxicity study.

Behavioral parameters	Acute toxicity
Eyes	Normal
Skin and Fur	Normal
Respiration	Normal
Salivation	Normal
Itching	Not found
Urine color	Normal
Feces consistency	Normal
Somatomotor activity	Normal
Sleep	Normal
Convulsions	Not found
Coma	Not found
Mortality	Not found

### Body and Organ Weights During Acute, Subacute, and Teratogenic Studies

**FIGURE 2:**
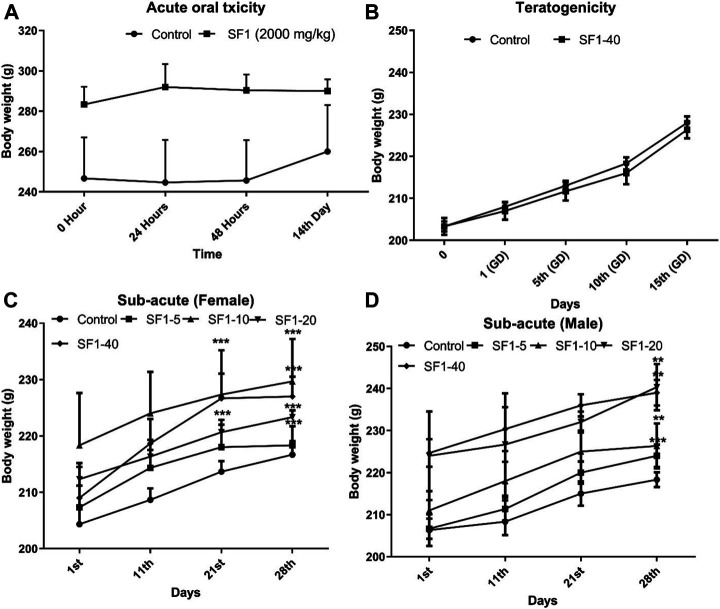
Effect of treatments on body weights during acute **(A)**, subacute **(C, D)**, and teratogenic toxicity **(B)** studies. Data is represented as mean ± SEM; *n* = 5 for acute and *n* = 10 for teratogenicity and subacute toxicity studies, *p < 0.05, **p < 0.01 and ***p < 0.001 when compared with day 1.

The variations in body weights and the weights of selected organs were observed during acute, subacute, and teratogenic studies. In acute toxicity study, normal increase in weight was seen in the treated rats with insignificant changes when compared to the control group, till the 14th day of observations. On the other hand, during the subacute study, a gradual and significant (*p* < 0.001) rise in the weights of male and female rats were observed throughout the subacute study in comparison to their weights on day-1 (Figure 2). During teratogenicity study, an increase in body weight was observed from the first gestational day (GD) to 15th GD, but this increase was almost parallel to the control of pregnant females (Figure 2). In case of the weights of the selected organs, each study showed an insignificant rise in the major organ weights except teratogenicity that showed a significant rise in the weight of the liver that may be attributed to the pregnancy (Table 2).

**TABLE 2:** Variations in organ weights of the control and treated animals during acute, subacute, and teratogenic studies.

	Dose	Organ mass (g)								
	(mg/kg)	Brain	Heart	Spleen	Kidney	Lungs	Liver	Stomach	Ovaries	Testis
Acute toxicity	Control	1.5 ± 0.2	0.3 ± 0.1	0.2 ± 0.1	1.2 ± 0.1	0.7 ± 0.3	6.9 ± 0.4	0.9 ± 0.8	0.2 ± 1.5	2.2 ± 1.2
	2000	1.45 ± 0.2	0.54 ± 0.1	0.41 ± 0.8	1.05 ± 0.1	0.92 ± 0.3	6.4 ± 0.5	1.1 ± 1.2	0.1 ± 0.9	2.6 ± 1.4
Subacute toxicity (male)	Control	1.3 ± 0.3	0.5 ± 0.2	0.3 ± 0.4	1.2 ± 0.3	0.8 ± 0.5	5.8 ± 0.5	1.1 ± 0.5		2.6 ± 0.4
	5	1.2 ± 0.1	0.6 ± 0.2	0.5 ± 0.3	1.2 ± 0.2	0.8 ± 1.3	6.9 ± 0.1	1.2 ± 0.0	-	2.2 ± 0.3
	10	1.4 ± 0.2	0.5 ± 0.3	0.4 ± 0.4	1.1 ± 0.8	0.8 ± 0.6	5.3 ± 1.2	1.6 ± 0.9	-	2.2 ± 0.5
	20	1.4 ± 0.6	0.5 ± 0.8	0.3 ± 03	1.4 ± 0.5	0.7 ± 0.7	6.4 ± 0.3	1.6 ± 0.8	-	2.1 ± 0.7
	40	1.2 ± 0.6	0.6 ± 0.5	0.4 ± 0.3	1.1 ± 0.1	0.8 ± 0.6	5.6 ± 0.2	1.9 ± 0.3	-	2.1 ± 0.1
Subacute toxicity (female)	Control	1.5 ± 0.4	0.6 ± 0.3	0.4 ± 0.1	1.1 ± 0.5	0.9 ± 0.2	6.1 ± 0.4	1.2 ± 0.3	0.1 ± 0.1	
	5	1.6 ± 0.6	0.9 ± 0.3	0.5 ± 0.3	1.4 ± 0.2	0.7 ± 0.1	6.6 ± 0.3	1.2 ± 0.5	0.1 ± 1.2	-
	10	1.5 ± 0.2	0.6 ± 0.1	0.4 ± 0.2	1.2 ± 0.1	0.7 ± 0.2	6.2 ± 0.3	1.1 ± 0.5	0.1 ± 0.1	-
	20	1.1 ± 0.3	0.6 ± 0.2	0.4 ± 0.3	0.9 ± 0.5	0.8 ± 0.3	4.9 ± 0.6	1.5 ± 0.7	0.1 ± 0.1	-
	40	1.1 ± 0.1	0.65 ± 0.3	0.3 ± 0.5	1.3 ± 0.3	0.8 ± 0.8	6.7 ± 0.5	1.6 ± 0.8	0.2 ± 0.9	-
Teratogenicity	Control	1.4 ± 0.2	0.62 ± 0.3	0.54 ± 0.1	1.2 ± 0.1	0.75 ± 0.2	6.24 ± 1.2	1.2 ± 0.2	0.32 ± 0.4	
	40	1.54 ± 0.1	0.55 ± 0.3	0.53 ± 0.4	1.23 ± 0.1	0.16 ± 0.5	11.17 ± 1.3^*^	0.9 ± 0.6	0.23 ± 0.3	-

Data represented as mean ± SEM with n = 5 for acute and n = 10 for teratogenicity and subacute toxicity studies. ^*^p < 0.05 was given in comparison to the control.

### Fetal and Placental Weights and Morphological Anomalies in Teratogenic Studies

**FIGURE 3:**
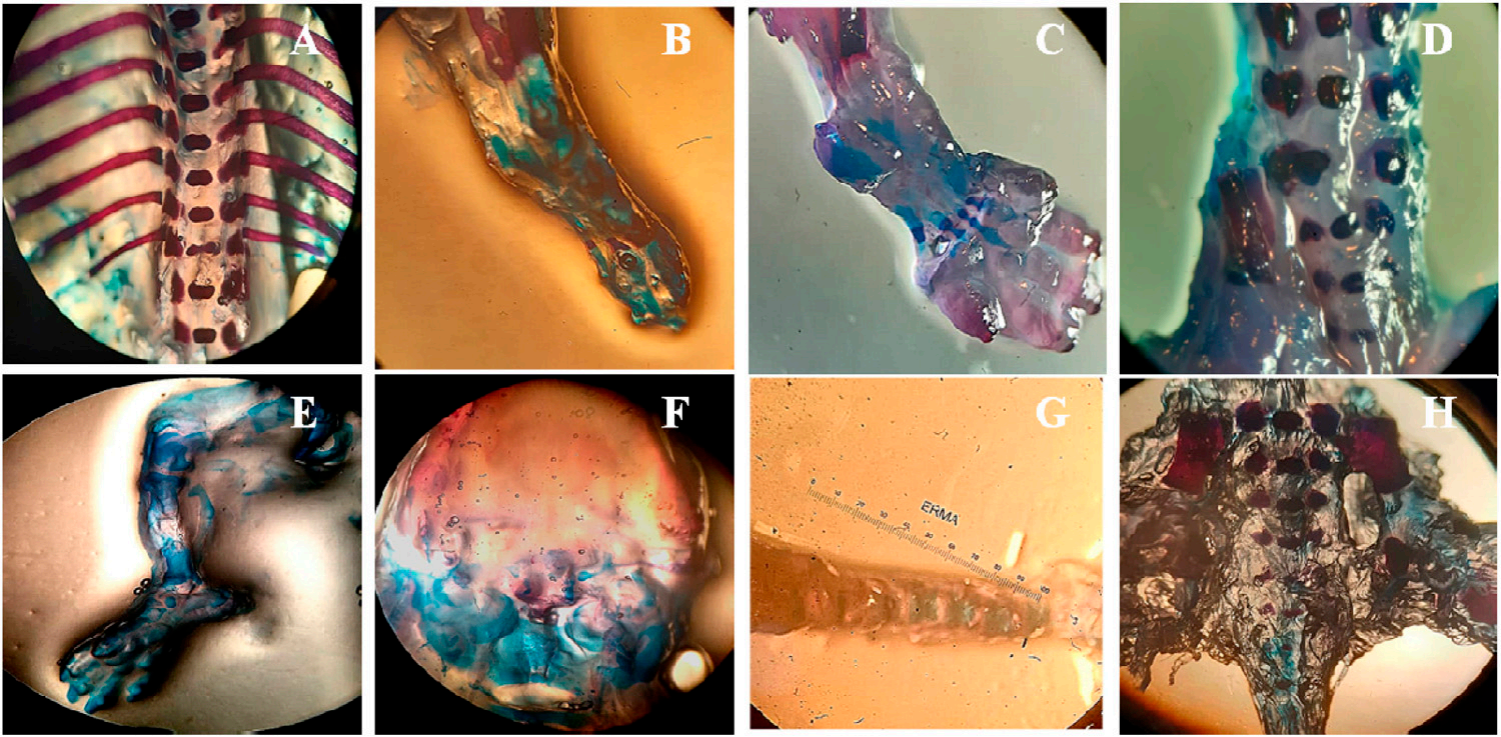
Effect of SF1 treatment (40 mg/kg) on skeletal anomalies in the teratogenic study. **(A)**: no ribs fusion, **(B)**: ossified hind limb bone, **(C)**: ossified bone of forelimb, **(D)**: normal legs bone, **(E)**: palate bone, **(F)**: normal cleft palate, **(G)**: tail bone, **(H)**: lower vertebral column.

Fetuses and placentas were removed carefully, and their weights were observed. Significant reduction in fetal and placental weights was observed in the treated animals (*p* < 0.05) while all other morphological and skeletal anomalies were absent in the treated group including the number of the alive and dead fetus, rib malformations, spina bifida, cleft palate, resorptions, and abortions (Table 3; Figure 3).

**TABLE 3:** Fetal and placental weights and morphological anomalies during the teratogenic study.

Anomalies	Group	
	Normal control	Treated group (40 mg/kg)
Cleft palate	0.0 ± 0.0	0.0 ± 0.0
Spina bifida (microns)	40 ± 0.3	42 ± 0.5
Rib malformation	0.0 ± 0.0	0.0 ± 0.0
Delayed cervical ossification	0.0 ± 0.0	0.0 ± 0.0
Early resorption	0.0 ± 0.0	0.0 ± 0.0
Late resorptions	0.0 ± 0.0	0.0 ± 0.0
Abortions	0.0 ± 0.0	0.0 ± 0.0
No. of litters	09 ± 0.3	07 ± 0.4
No. of live fetuses	08 ± 0.3	05 ± 0.4
Maternal death rate	01 ± 0.1	02 ± 0.2
Fetal weight (gm)	5.3 ± 0.1	3.2 ± 1.3^*^
Placental weight (gm)	0.7 ± 0.2	0.55 ± 0.2^*^

The is data represented as mean ± SEM (n = 10). *p < 0.05 was given in comparison to the control.

## Soft Tissue Anomalies in Teratogenic Studies

**FIGURE 4:**
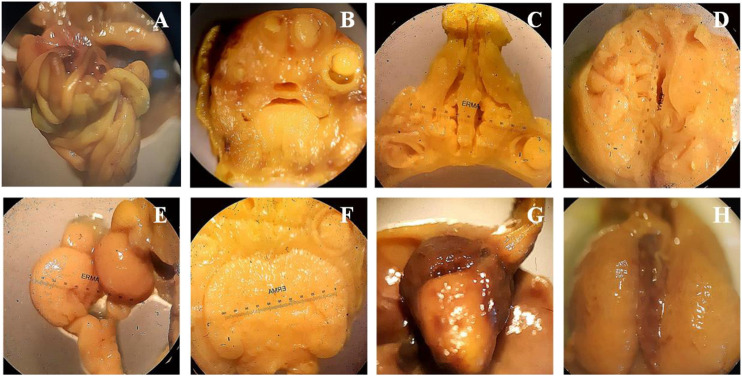
Wilson’s technique for soft tissue examination. **(A)**: intestine, **(B)**: frontal Head section, **(C)**: palate section, **(D)**: no hydrocephalus, **(E)**: kidney, **(F)**: 3rd lateral ventricle, **(G)**: heart, **(H)**: liver.

Bouins fixation method was used for the assessment of soft tissues anomalies during teratogenicity assessment, including the observation of hydrocephalus and hydronephrosis. Treatment of SF1 (40 mg/kg) did not induce any abnormality in the soft tissues along with no observable hydrocephalus and hydronephrosis (Figure 4).

### Morphology and Concentration of Sperms in Subacute Study

**FIGURE 5:**
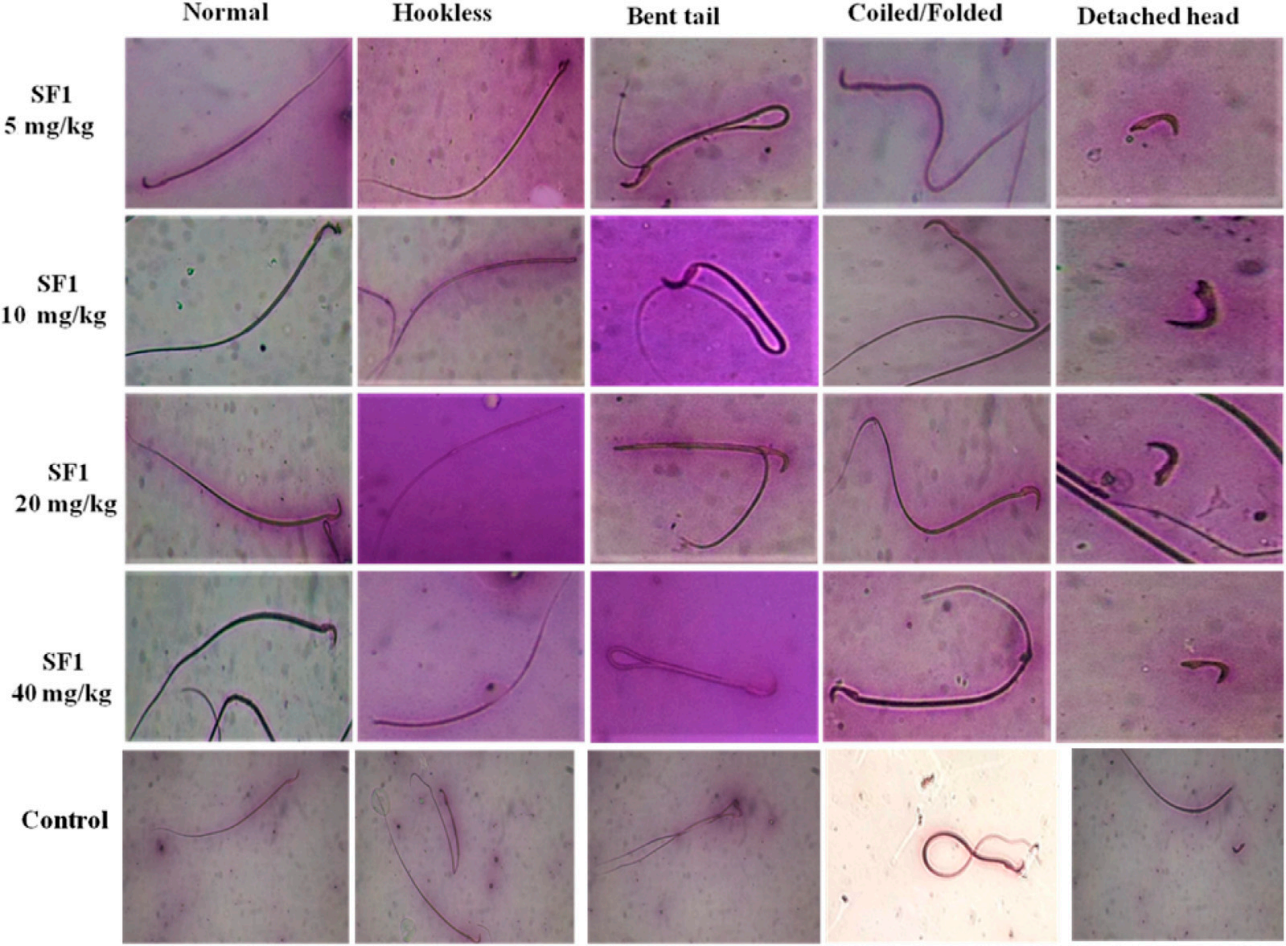
Pictorial representation of sperm morphology (bent tail, coiled and detached head) after treatment with different SF1 dose levels in subacute toxicity study.

Semen was collected from cauda epididymis to examine the sperm morphology after 28 days of treatment. A dose of 5 mg/kg SF1 significantly increased the sperm count and with the reduction in the percentage of hookless, bent and detached head sperms, in comparison to the control group (Figure 5). A higher dose of 40 mg/kg presented a reduction in sperm count with no significant deviation in morphological parameters as compared to the control (Table 4).

**TABLE 4:** Sperm count and morphology of SF1 treated animals (male) at different dose levels in subacute toxicity study.

Parameters	Groups (mg/kg)				
	Control	SF1			
	1	5	10	20	40
Sperm count (×106 sperm/mL)	55.5 ± 2.1	170.4 ± 3.2^***^	56.3 ± 1.2	72.4 ± 2.2^**^	42.7 ± 2.4
Normal sperm (%)	74.5	90.2^***^	72.3	76.45	68.53
Hock less (%)	0.98	0.47^***^	0.62^*^	1.1	0.89
Bent (%)	5.5	2.2^***^	3.2^*^	6.5	5.9
Coiled/folded (%)	8.6	7.3	6.4	6.9	5.4
Detached head (%)	10	15^*^	30^***^	18*	11

The control used is CMC. Data represented as mean ± SEM (n = 5). ^*^p < 0.05,^**^p < 0.01 and^ ***^p < 0.001 were given in comparison to the control.

### Hematological Parameters in Acute, Subacute, and Teratogenic Studies

At the end of each study, blood samples were collected by the cardiac puncture, and complete blood count (CBC) was performed. Results showed that WBC count was raised significantly in acute toxicity and teratogenicity regimes and in females of subacute toxicity studies (*p* < 0.05) when compared to the control group. RBC count was normal in all treatment studies. Platelet count was decreased significantly (*p* < 0.001) in acute and subacute studies; however, a significant (*p* < 0.001) rise was seen during teratogenicity evaluation when compared with the control group. No deviations were observed in the levels of HB, MCH, MCHC, MCV, and HCT, in comparison to the control group. A significant elevation was observed in the levels of lymphocytes in all studies in comparison to the control group except male rats at the dose levels of 5 and 10 mg/kg, where the values stayed close to the normal. In acute and subacute (male) toxicity studies significant changes in the level of granulocytes were also analyzed in comparison to control (Table 5).

**TABLE 5:** Effect of different studies (acute toxicity, subacute toxicity, and teratogenicity) on hematological parameters.

Treatment (mg/kg)	WBC	RBC	Platelets	HB	Lym	Mid	Gra	MCH	MCHC	MCV	HCT
	(×10^3^/µl)	(×10^6^/µl)	(×10^3^/µl)	(g/dl)	(10^3^/µl)	(10^3^/µl)	(10^3^/µl)	(pg)	(g/dl)	(fL)	(%)
Control	3.7 ± 0.1	5.9 ± 0.3	817 ± 0.1	12.0 ± 1.2	3.1 ± 1.4	0.4 ± 1.3	0.1 ± 0.02	20.3 ± 0.1	36.5 ± 0.9	55.4 ± 1.2	33.0 ± 3.2
Acute oral toxicity											
SF1 (2000)	17.5 ± 1.2^***^	7.95 ± 3.2	474 ± 0.21^^^^^	14.2 ± 0.014	15.2 ± 1.08^**^	0.4 ± 0.5	1.3 ± 0.32^**^	18.10 ± 0.23	33.1 ± 0.85	54.7 ± 0.2	43.5 ± 1.3
Subacute toxicity (male)											
Control	4.5 ± 0.1	6.1 ± 0.3	745 ± 0.14	12.6 ± 1.2	3.1 ± 1.4	0.9 ± 1.3	0.1 ± 0.02	20.30 ± 0.15	36.5 ± 0.96	55.4 ± 1.2	33.0 ± 3.2
SF1 (5)	4.6 ± 0.42	6.12 ± 0.38	459 ± 0.43^^^^^	12.9 ± 0.01	2.9 ± 1.0	0.7 ± 0.62	1.0 ± 0.01^**^	21.0 ± 0.31	35.5 ± 0.78	59.1 ± 0.2	36.2 ± 1.3
SF1 (10)	4.2 ± 0.21	6.13 ± 0.16	615 ± 0.21	13.0 ± 0.12	3.0 ± 1.1	0.8 ± 0.7	0.4 ± 0.12	21.2 ± 0.25	36.2 ± 0.82	58.6 ± 0.1	35.6 ± 1.5
SF1 (20)	4.0 ± 0.43	4.94 ± 1.2	417 ± 0.12^^^^^	10.9 ± 0.11	85.8 ± 0.98^***^	6.3 ± 0.5^***^	7.9 ± 0.13^***^	20.1 ± 0.2	34.9 ± 0.84	57.7 ± 0.1	22.7 ± 1.1
SF1 (40)	5.0 ± 0.15	5.89 ± 1.1	350 ± 0.32^^^^^	10.6 ± 1.1	91.8 ± 1.02^***^	5.4 ± 0.6^***^	2.8 ± 0.1^**^	18 ± 0.12	34.4 ± 0.79	52 ± 0.2	30.9 ± 2.4
Subacute toxicity (female)											
Control	5.5 ± 0.12	6.0 ± 0.3	735 ± 0.1	11.2 ± 1.2	2.9 ± 1.4	0.6 ± 1.3	0.1 ± 0.02	20.3 ± 0.1	36.5 ± 0.9	55.4 ± 1.2	33.0 ± 3.2
SF1 (5)	10.8 ± 0.2^***^	5.92 ± 1.1	221 ± 0.2^^^^^	12.7 ± 0.1	9.6 ± 1.0^*^	0.8 ± 0.3	0.4 ± 0.0	21.6 ± 0.1	34.1 ± 0.8	63.3 ± 0.1	37.5 ± 1.2
SF1 (10)	10.2 ± 0.1^***^	5.11 ± 1.2	750 ± 0.3	11.2 ± 0.1	9.0 ± 1.1^*^	0.8 ± 0.5	0.4 ± 0.2	22 ± 0.2	36.1 ± 0.8	61.0 ± 0.2	31.2 ± 1.5
SF1 (20)	9.0 ± 0.3^***^	6.53 ± 2.1	188 ± 0.1^^^^^	13.5 ± 0.1	7.3 ± 1.0^*^	1.1 ± 0.6	0.6 ± 0.6	20.6 ± 0.2	35.9 ± 0.9	57.5 ± 0.1	37.5 ± 1.1
SF1 (40)	11.8 ± 0.2^***^	6.55 ± 1.3	606 ± 0.3	12.9 ± 0.1	10.1 ± 1.1^*^	1.1 ± 0.6	0.6 ± 0.1	19.9 ± 0.2	35.1 ± 0.7	56.7 ± 0.2	37.1 ± 1.2
Teratogenicity											
Control	5.7 ± 0.1	5.95 ± 0.3	821 ± 0.1	11.5 ± 1.2	6.1 ± 1.4	0.5 ± 1.3	0.1 ± 0.02	20.3 ± 0.1	36.5 ± 0.9	55.4 ± 1.2	33.0 ± 3.2
SF1 (40)	12.8 ± 0.2^***^	5.65 ± 1.1	1,000 ± 0.2^***^	11.0 ± 0.14	10.9 ± 1.1^*^	1.5 ± 0.5	0.4 **±** 0.1	19.6 ± 0.2	33.5 ± 0.8	58.6 ± 0.2	33.1 ± 1.2

Data represented as mean ± SEM; n = 5 for acute toxicity and n = 10 for teratogenicity and subacute toxicity studies. ^*^p < 0.05, ^**^p < 0.01, ^***^p < 0.001 were increased levels of significance while ^^^^^ p < 0.001 was a decreased level of significance in comparison to the control.

### Biochemical Parameters in Acute, Subacute, and Teratogenic Studies

A panel of biochemical parameters was performed at the end of acute and subacute toxicity studies to assess the effects of SF1 on liver, kidney, and lipid profile. For the liver function test, AST, ALP, ALT, and bilirubin were measured while creatinine, uric acid and total protein were performed to examine kidney health. In the acute study, ALP and bilirubin levels were significantly (*p* < 0.05) raised in comparison to control. At the same time, all other parameters for the lipid profile and kidney function test were normal and within the range (Table 6).

**TABLE 6:** Estimation of biochemical markers in acute oral toxicity.

Biochemical markers	Control	SF1
Uric acid (mg/dl)	5.0 ± 1.2	1.46 ± 0.23
Protein (g/dl)	7.65 ± 1.2	8.7 ± 1.53
Creatinine (mg/dl)	1.12 ± 2.1	1.00 ± 0.52
Bilirubin (mg/dl)	1.2 ± 1.42	3.73 ± 0.63^a^
ALP (U/L)	185 ± 1.97	394.5 ± 0.33^a^
ALT (U/L)	42 ± 0.34	39.2 ± 2.59
AST (U/L)	65 ± 1.45	71.5 ± 1.06
Urea (mg/dl)	30.2 ± 0.12	33.5 ± 1.02
Cholesterol (mg/dl)	65.12 ± 2.1	50.1 ± 0.89
HDL (mg/dl)	20.3 ± 1.23	13.2 ± 0.54
Triglyceride (mg/dl)	56.84 ± 1.2	59.23 ± 0.54
LDL (mg/dl)	33.32 ± 0.32	25.2 ± 1.45

The data represented as mean ± SEM (n = 6).

^a^ p < 0.05 was increase level given in comparison to the control.

**FIGURE 6:**
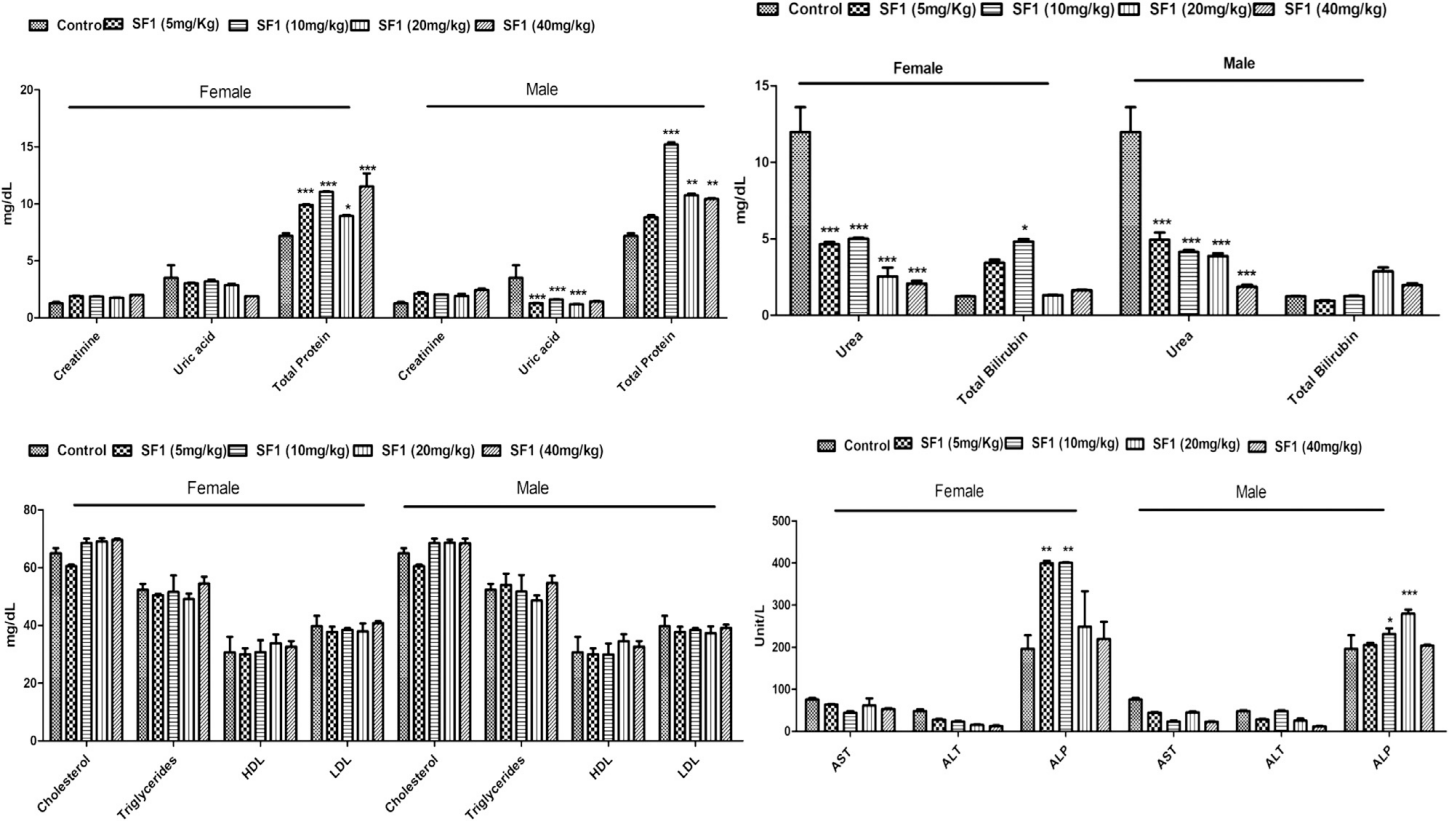
Estimation of biochemical parameters after 28 days of treatment with different dose levels of SF1 in the subacute study. Data represented as mean ± SEM (*n* = 10). *p < 0.05, **p < 0.01 and ***p < 0.001 was in comparison to the control.

In the subacute study, liver parameters, kidney functioning tests, and lipid profile were measured in both male and female treated rats. In drug-treated female rats, ALP was raised significantly at 5 and 10 mg/kg dose levels while in the male, it was raised by 10 and 20 mg/kg doses. Levels of AST were increased at 10 and 40 mg/kg doses in treated male rats. Total protein levels were increased in both male and female on almost all dose levels. No effect was seen in any parameter of lipid profile, creatinine, and total bilirubin levels in both male and female rats. Treated male and female rats presented significant (*p* < 0.001) reduction in the uric acid levels (Figure 6).

### Effect of Acute and Subacute Toxicity Studies on Thyroid Functioning

Any abnormality in the thyroid functioning was assessed by estimating the T_3_, T_4_, and TSH levels in acute and subacute toxicity studies (Table 7). No significant changes were observed in the levels of T_3_, T_4_, and TSH in both acute and subacute toxicity studies when compared to the control group.

**TABLE 7:** Estimation of thyroid function markers in treated groups during acute and subacute toxicity studies.

Parameters	Control	Acute toxicity (2000 mg/kg)	Subacute toxicity (40 mg/kg)				
			Control	Male	Control	Female	
T3 (ng/dl)	46.2 ± 1.6	50.23 ± 1.3	42.3 ± 1.6	46.58 ± 1.4	47.3 ± 1.7	48.58 ± 0.9	
T4 (µg/dl)	4.9 ± 1.2	5.63 ± 0.2	5.2 ± 1.6	5.1 ± 0.5	4.6 ± 0.5	4.07 ± 0.7	
TSH (µIU/ml)	0.037 ± 0.32	0.01 ± 0.05	0.05 ± 0.01	0.06 ± 0.032	0.06 ± 0.01	0.083 ± 0.02	

The data is represented as mean ± SEM with n = 5 for acute and n = 10 for subacute toxicity studies.

### Effect of Acute, Subacute, and Teratogenic Studies on Oxidative Stress Biomarkers

Different oxidative stress markers (SOD, CAT, GSH, and MDA) were estimated at the end of each study for analyzing any toxicity at the tissue level. In acute oral toxicity study in kidney levels of SOD, GSH, and MDA were significantly decreased in comparison to control whereas in brain, stomach, and spleen GSH levels were raised considerably (*p* < 0.05). Reduced levels of CAT were found to be in the brain, lungs, and ovarian tissues. In teratogenicity no significant difference was seen between control and treated groups in levels of SOD, CAT, and GSH however a substantial rise in MDA levels were observed in major organs like kidney, spleen, brain, and lungs (Table 8).

**TABLE 8:** Effect of SF1 treatment on oxidative stress biomarkers of selected organs during acute oral toxicity and teratogenicity study.

Oxidative stress markers	Toxicity	Treatment	Organs							
			Liver	Kidney	Brain	Stomach	Spleen	Lungs	Testis	Ovaries
SOD (µg/mg of tissue protein)	Acute oral toxicity	Control	9.9 ± 0.5	28.7 ± 0.4	28.6 ± 0.5	18.23 ± 0.3	20.3 ± 2.3	19.2 ± 0.7	28.5 ± 0.5	21.5 ± 0.12
		SF1	6.2 ± 1.23	12.6 ± 0.4^^^	21.8 ± 0.9	24.0 ± 1.3*	30.2 ± 0.2	25.0 ± 1.0^*^	25.5 ± 0.3	19.4 ± 1.2
	Teratogenicity	Control	8.9 ± 0.5	20.7 ± 0.6	25.2 ± 0.1	16.3 ± 0.4	24.2 ± 1.3	20.1 ± 0.7	-	20.6 ± 1.12
		SF1	8.6 ± 1.3	16.2 ± 1.2^	22.3 ± 2.1	20.5 ± 0.6	25.6 ± 0.3	18.2 ± 2.1	-	22.3 ± 2.1
GSH (µg/mg of tissue protein)	Acute oral toxicity	Control	0.14 ± 0.1	2.05 ± 0.11	0.133 ± 0.11	1.32 ± 2.1	1.56 ± 1.2	2.89 ± 0.1	0.21 ± 0.3	0.13 ± 0.1
		SF1	0.7 ± 0.5^*^	0.83 ± 1.0^^^	1.37 ± 0.32^*^	2.98 ± 0.87^*^	2.63 ± 1.2*	1.00 ± 0.7^^^	0.13 ± 0.6	0.76 ± 0.2^*^
	Teratogenicity	Control	0.24 ± 0.4	1.9 ± 0.1	0.83 ± 0.11	1.22 ± 2.1	1.66 ± 1.8	1.89 ± 0.2	-	0.43 ± 0.1
		SF1	0.23 ± 0.1	1.2 ± 0.54	0.97 ± 0.41^^^	1.4 ± 0.32	1.64 ± 0.3	1.5 ± 0.11	-	0.95 ± 0.6^*^
CAT (mmole/min/mg of protein)	Acute oral toxicity	Control	2.03 ± 0.7	4.53 ± 0.54	6.0 ± 2.63	3.35 ± 1.5	2.13 ± 3.2	7.5 ± 0.68	1.02 ± 3.1	4.35 ± 3.2
		SF1	2.65 ± 1.3	5.03 ± 0.65	3.09 ± 1.32^^^	4.71 ± 0.63	1.63 ± 0.5	3.6 ± 0.7^	0.98 ± 1.2	2.3 ± 1.5^
	Teratogenicity	Control	3.03 ± 0.9	5.53 ± 0.4	5.90 ± 2.6	4.35 ± 1.5	1.93 ± 3.2	6.2 ± 0.68	-	3.35 ± 3.2
		SF1	3.56 ± 0.3	4.98 ± 0.5	5.32 ± 1.2	4.25 ± 1.4	1.98 ± 3.2	6.54 ± 1.2	-	3.59 ± 0.65
MDA (µmole/mg of tissue protein)	Acute oral toxicity	Control	0.04 ± 0.0	2.0 ± 0.01	1.58 ± 0.01	0.12 ± 0.3	0.025 ± 0.3	2.75 ± 0.01	0.54 ± 0.2	1.5 ± 0.3
		SF1	0.03 ± 1.0	0.04 ± 0.6^	0.02 ± 1.52^^^	0.1 ± 0.87	0.24 ± 0.32	0.30 ± 0.52^^^	0.12 ± 0.01^^^	0.78 ± 1.2^^^
	Teratogenicity	Control	0.03 ± 0.1	1.05 ± 0.02	1.38 ± 0.01	0.12 ± 0.3	0.15 ± 0.3	1.85 ± 0.01	-	1.5 ± 0.3
		SF1	0.05 ± 0.2	1.2 ± 0.2^^^	0.01 ± 0.9^^^	0.99 ± 0.75^*^	0.12 ± 0.41	1.65 ± 0.5	-	0.63 ± 1.1^^^

Data represented as mean ± SEM (n = 3). ^*^p < 0.05 and ^^^fn^ p < 0.05 were increased and decreased levels of significance, respectively, in comparison to the control.

**FIGURE 7:**
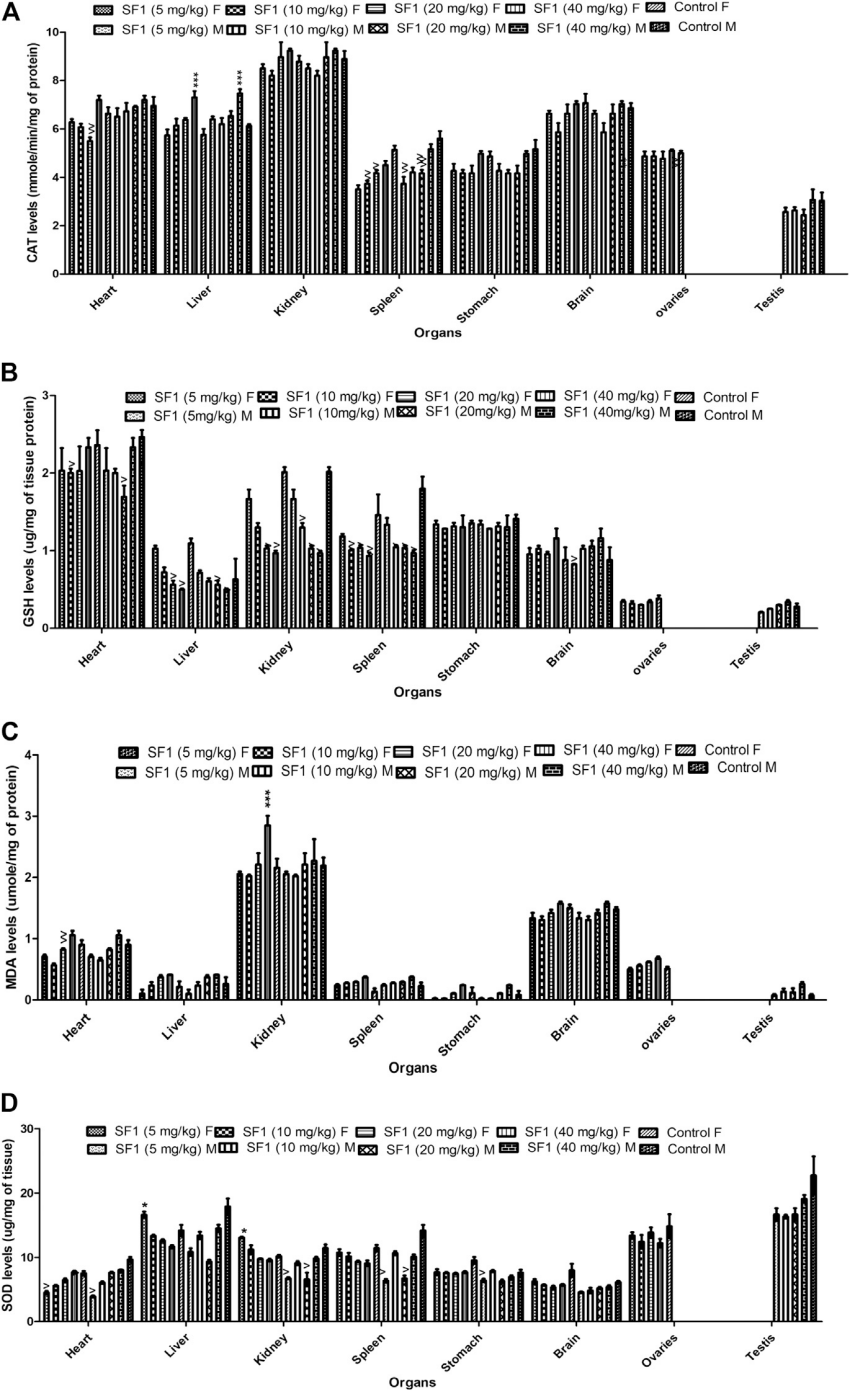
Estimation of oxidative stress markers in the subacute toxicity study in male and female rats. The data is presented as mean ± SEM (*n* = 3). *p < 0.05 and ^*p* < 0.05 were increased and decreased levels of significance, respectively, in comparison to the control. M = Male, F = Female.

Females were more prone to the toxicity as compared to the male. In subacute toxicity studies levels of the antioxidants like SOD, CAT, and GSH were decreased in kidney and spleen tissues of male rats. Heart and liver were also affected as SOD levels were reduced in the liver at lower doses, but it raised when treated with high doses while GSH levels were significantly decreased in the heart of male rats. In male rats, MDA levels seem to be normal in almost all tissues when compared with control (Figure 7). Results showed that in female rats, levels of GSH were declined in liver, kidney, and spleen tissues at higher doses (20 and 40 mg/kg) in comparison to control group. CAT and SOD levels were also affected in spleen and liver tissues of female rats.

### Histopathological Analysis

**FIGURE 8:**
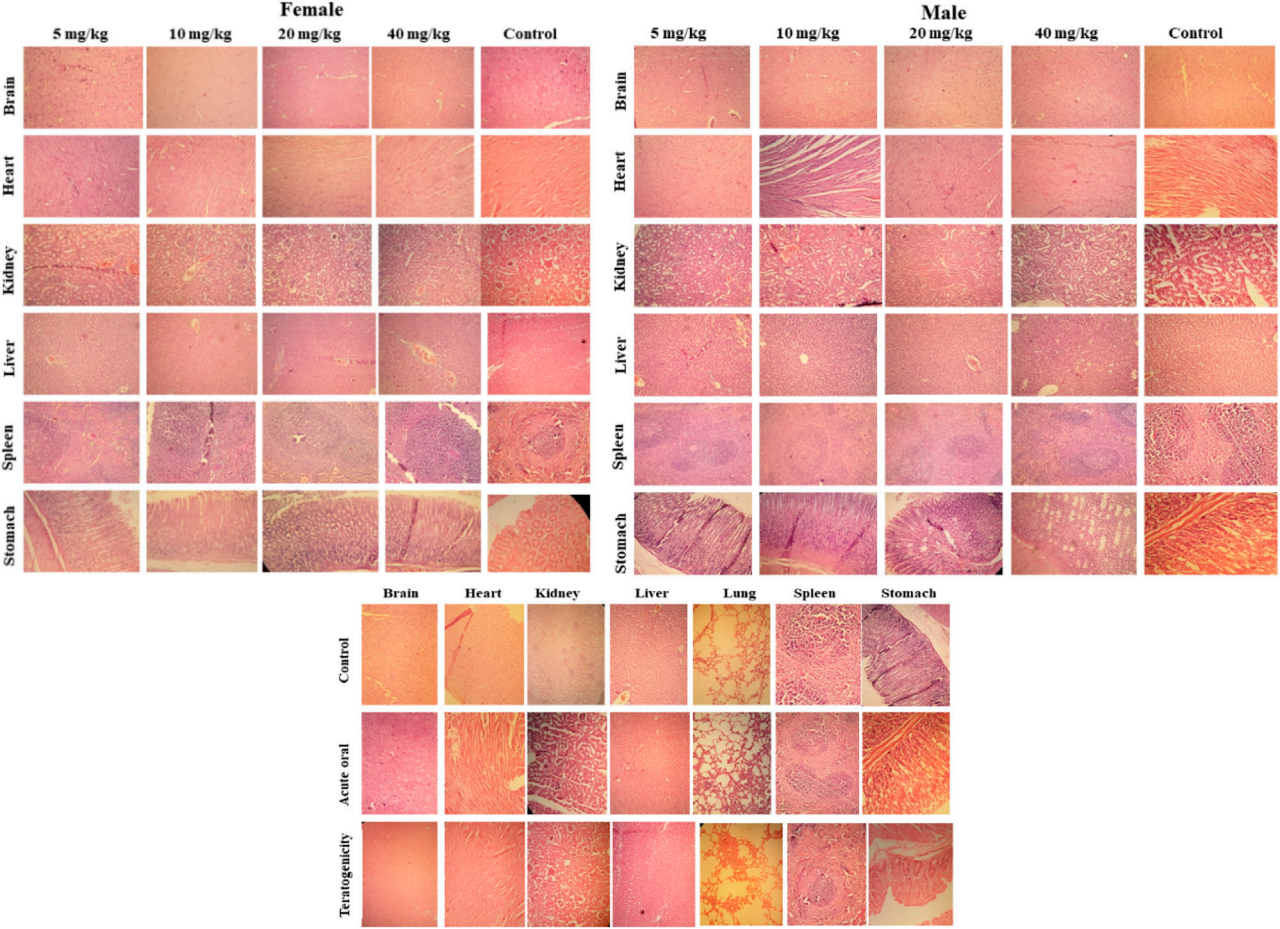
Histopathological examination of selected tissues (using hematoxylin and eosin staining) of rats treated with SF1 in subacute toxicity (at 5, 10, 20 and 40 mg/kg), acute oral toxicity (2000 mg/kg), and teratogenicity (40 mg/kg). The results are presented in comparison to the control in all three dose regimes.

Histopathology revealed that all treated groups after 14th days (acute oral toxicity), 19th day (teratogenicity), and 28th days (Subacute) showed no cellular changes in their major organs in comparison to the control group. All the organs showed normal architecture (Figure 8).

### ELISA Analysis

**FIGURE 9:**
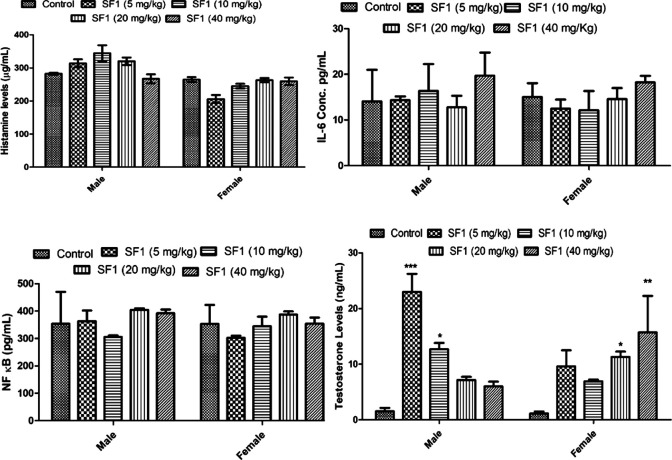
Estimation of histamine, testosterone, IL-6 and NF-κB levels in serum during subacute toxicity study. The data is represented as mean ± SEM (*n* = 3). *p < 0.05 is the significance level in comparison to the control.

Levels of testosterone, histamine, IL-6 and NF-κB were estimated in the subacute study. The results showed that SF1 significantly elevated the levels of testosterone both in male, as well as female when compared with control. Histamine, IL-6, and NF-κB remained normal and within the range in all treated groups (Figure 9).

## Discussion

Acute and chronic deleterious effects of chemicals and drugs have become an issue of great interest for the scientists. Different laws are in place in different countries that provide directions to the scientists, manufacturers, and distributors for the toxicity profiling of the molecules. These laws and guidelines regulate, as well as advocate the toxicological profiling of the development of the molecule of strict rules and regulations and increasing concern regarding the safety of human health has made toxicological profiling a markedly expanded field. The toxicological profiling includes the single and repeated administration of the test compounds in animals and demands special investigations on male and female animals (Rand and Petrocelli, 1985).

Initially, the primary purpose of this study was to a singly highest dose that could induce significant adverse effects or life-threatening toxicity (Klaassen and Amdur, 2013). An acute toxicity study was carried out at a single dose of 2000 mg/kg SF1, administered orally. No mortality or life-threatening toxicity was observed until the 14th day of treatment, indicating that *LD*_*50*_ of SF1 is significantly higher than 2000 mg/kg. In common practice, a change in the body weight pattern and general behavior is preliminary indicators of the adverse effects of the chemicals (Variya et al., 2019). In acute toxicity study, any change in the body weight and general behavior was observed from the day 1st to 14th day, and results showed an insignificant difference in body weight and significant difference in organ weight (Figure 2).

In SF1, the ester linkage is possibly most susceptible to dissociation in the biological system. For example, in the case of oral administration, stomach acid could catalyze ester hydrolysis resulting in the formation of 2-naphthol and 3,5-dinitrobenzoic acid. The *LD*_*50*_ values for oral administration of 2-naphthol and 3,5-dinitrobenzoic acid in rats are 1960 and 860 mg/kg, respectively. It is evident from the current study that an oral dose of 2000 mg/kg SF1 induced no considerable toxicity indicating that its ester bond is not considerably prone to hydrolysis. This might be due to the lipophilic nature of SF1 and the presence of bulky aromatic residues that could hinder the approach of water for hydrolysis and proton attach on ester moiety, thus, avoiding acid-induced ester hydrolysis. Furthermore, many marketed drugs having aromatic and aliphatic esters bonds are administered orally, e.g., aspirin, propanidid, salsalate, remimazolam, etc. Additionally, many studies are available with oral administration of compounds closely related to SF1 via oral route for *in vivo* studies (Husain et al., 2012; Abbas et al., 2010; Masic et al., 2015).

Subacute toxicity study was carried out to find out any abnormality in the organs after the repeated administration of the test compound at different dose levels (Abubakar et al., 2019; Anwar et al., 2020). In this study, four doses of SF1 (5, 10, 20, and 40 mg/kg) were selected for the subacute toxicity study. In our previous study, these dose levels depicted a considerable reduction in AChE activity in the rat brain. Each group received their respective treatments for 28 days consecutively. Body weights were significantly increased from day 1 to day 28 of treatments in both male and female rats. However, the non-significant difference was observed in all selected organs weights in comparison to control.

Hematological and biochemical parameters are *bona fide* indicators of the toxicity and organ dysfunction (Adeneye et al., 2006). Overall, female rats were more sensitive toward deviation in biochemical parameters as compared to the male. All the blood cells are obtained from the pluripotent stem cells that become mature and differentiate into the RBC, WBC and platelets (Ugwah-Oguejiofor et al., 2019). WBC and differential leukocyte count levels were raised in acute oral, subacute (female) and in teratogenicity in comparison to control indicating the boost up in the immune system (Mythilypriya et al., 2007). This increase might be due to the immunopotentiation effect of the SF1. The significant alteration was observed in the levels of platelets during acute and subacute male toxicity studies, but this reduction is within the range. All other parameters of hematology were normal and close to the control. The estimation of biochemical parameters for liver and kidney are considered primary organs responsible for metabolism and elimination of the circulating drug (Bariweni et al., 2018). Any alteration in the values of liver function parameters (ALP, AST, ALT, bilirubin, and total protein) represent hepatotoxic nature of the drug (Abdelkader et al., 2020). Levels of ALP were raised in acute and subacute (male and female) toxicity studies while all other biochemical parameters were normal and within the range.

Teratogenicity is of fundamental importance in the toxicological evaluation of new molecules because it reduces the births of malformed infants (Macklin, 2010). After the subacute study, the highest dose (40 mg/kg) was selected for the teratogenicity studies. In this study, morphological anomalies, soft tissues anomalies and any skeletal anomalies were detected by teratogenicity studies. Results showed that at 40 mg/kg dose level SF1 did not show any abnormality in the skeleton, soft tissues and morphology (Table 3; Figure 3, and Figure 4).

Hormonal imbalances and infertility induced by the direct or indirect exposure of a dug is termed as reproductive toxicity (de Barros et al., 2016). The evaluation of reproductive toxicity is mandatory to examine the effects of a putative new drug in an animal model (Fu et al., 2009; Karnam et al., 2015). In this study, change in the weight of testis and concentration and morphology of sperms was analyzed. Results showed that animals treated with the lowest dose of SF1 (5 mg/kg) induced spermatogenesis as sperm count was increased, while abnormal morphological changes were reduced significantly (Table 4). Sperm production in the testis is the important marker of male infertility. The test doses of SF1 higher than 5 mg/kg presented sperm count close to that of control. Sperm count increase by SF1 might be due to the progression of the spermatogenesis initiated by the testosterone, as SF1 significantly elevated the levels of testosterone in both male and female rats.

In living systems, a continuous exposure or production of reactive oxygen species (Zhang et al., 2020) may give rise to the disturbed redox homeostasis or oxidative stress that could damage proteins, nucleic acids and lipids (Jones, 2006). If the cell is burdened with oxidative stress, apoptosis, necrosis, and irreversible cell damage may occur. As a result, various cascade pathways of defense mechanism including gene controlling cycle, repair and different antioxidant pathways are triggered for the protection against oxidative stress (Nair et al., 2015). In the present study, endogenous antioxidants (SOD, CAT, and GSH) and oxidants (MDA) were estimated in the selected organs of the animals to access any imbalance and cell damage induced because of SF1 dosing. In acute oral toxicity study, a significant decrease in SOD and GSH levels were observed in the kidney, and the level of CAT was decreased in lungs. In teratogenicity study, SOD levels in the kidney and GSH and CAT levels in the brain were dropped. In subacute toxicity study, SOD, CAT, and GSH levels were decreased in the spleen of both male and female rats that might be due to the immunostimulatory effect of the drug as WBC levels were also increased. SF1 is a lipophilic compound, and it might be excreted out by the kidney, indicated by the decrease in levels of oxidative stress markers in both male and female rats.

Moreover, heart and brain tissues also showed a small reduction in stress markers. In histopathology, no change was observed in any organ at cellular levels. Oxidative stress plays a significant contributing role in the progression of inflammation (Chen et al., 2016). In the present study, different inflammatory mediators (histamine, IL-6 and NF-κB) were also estimated in the blood of the treated animals of subacute toxicity study. No increase was found in the levels of histamine, IL-6, and NF-κB levels. SF1 did not increase the inflammatory markers, biochemical markers and did not produce any toxic effects in the selected organs confirming its safety.

A detailed literature survey was carried out to find reported activities of the test compound using CHEMBL, PubChem, and SciFinder databases. It was observed that phenyl 3,5-dinitrobenzoate is an anti-inflammatory agent with *IC*_*50*_ of 15.8 µM for arachidonate 5-lipoxygenase inhibition in human whole blood assay (Shang et al., 2014). Moreover, a closely related derivative of SF1 without nitro groups (naphthalen-2-yl benzoate) has been reported as an inhibitor of tau fibril formation with 14.1 µM *IC*_*50*_ in CHEMBL database (CHEMBL1407906). Hence, these accounts from the literature corroborate the finding reported in this study.

## Conclusion

The *LD*_*50*_ of SF1 is higher than 2000 mg/kg, and no sign and symptoms of toxicity and mortality were reported. Prolonged and repeated dose administration at the different dose levels revealed that it might cause very mild liver toxicity due to the elevation of ALP levels. SF1 is safe for pregnancy as it showed no teratogenic toxicity. The estimation of biochemical, oxidative stress and anti-inflammatory markers did not show significant deviation as compared to the control. This study indicated that SF1 could be used for further pharmacological studies on the doses, as mentioned earlier. Mutagenicity and carcinogenicity studies will be performed in future.

## Data Availability

The raw data supporting the conclusions of this article will be made available by the authors, without undue reservation.

## References

[B1] Abbas S. Greige-Gerges H. Karam N. Piet M. H. Netter P. Magdalou J. (2010). Metabolism of parabens (4-hydroxybenzoic acid esters) by hepatic esterases and UDP-glucuronosyltransferases in man, Drug Metab. Pharmacokinet., 25, 568–577. 10.2133/dmpk.dmpk-10-rg-013 20930423

[B2] Abdelkader N. F. Elyamany M. Gad A. M. Assaf N. Fawzy H. M. Elesawy W. H. (2020). Ellagic acid attenuates liver toxicity induced by valproic acid in rats. J. Pharmacol. Sci. 143 (1), 23–29. 10.1016/j.jphs.2020.01.007 32139333

[B3] Abubakar A. Nazifi A. Hassan F. Duke K. Edoh T. (2019). Safety assessment of Chlorophytum alismifolium tuber extract (Liliaceae): acute and sub-acute toxicity studies in Wistar rats. J. Acute Dis. 8 (1), 21. 10.4103/2221-6189.250374

[B4] Adeneye A. A. Ajagbonna O. P. Adeleke T. I. Bello S. O. (2006). Preliminary toxicity and phytochemical studies of the stem bark aqueous extract of Musanga cecropioides in rats. J. Ethnopharmacol. 105 (3), 374–379. 10.1016/j.jep.2005.11.027 16413715

[B5] Aneela S. De S Kanthal L K Choudhury N.S.K Sagar K V Das B L (2011). Acute oral toxicity studies of Pongamia Pinnata and Annona squamosa on albino wister rats. Int. J. Res. Pharm. Chem. 1 (4), 820–824.

[B6] Anwar F. Saleem U. Ahmad B. Ashraf M. Rehman A. U. Froeyen M. (2020). New naphthalene derivative for cost-effective AChE inhibitors for Alzheimer’s treatment: in silico identification, *in vitro* and *in vivo* validation. Comput. Biol. Chem. 89, 107378. 10.1016/j.compbiolchem.2020.107378 33002716

[B7] Arome D. Chinedu E. (2013). The importance of toxicity testing. J. Pharm. BioSciences 4, 146–148.

[B8] Bariweni M. W. Yibala O. I. Ozolua R. I. (2018). Toxicological studies on the aqueous leaf extract of Pavetta crassipes (K. Schum) in rodents. J. Pharm. Pharmacogn. Res. 6 (1), 1–16.

[B9] Barrow M. V. Taylor W. J. (1969). A rapid method for detecting malformations in rat fetuses. J. Morphol. 127 (3), 291–305. 10.1002/jmor.1051270303 4388962

[B10] Buschmann J. (2013). The OECD guidelines for the testing of chemicals and pesticides. Methods Mol. Biol., 947, 37–56. 10.1007/978-1-62703-131-8_4 23138894

[B11] Cartner S. C. Barlow S. C. Ness T. J. (2007). Loss of cortical function in mice after decapitation, cervical dislocation, potassium chloride injection, and CO2 inhalation. Comp. Med. 57 (6), 570–573. 18246869

[B12] Chen R. Zhao L. Bai R. Liu Y. Han L. Xu Z. (2016). Silver nanoparticles induced oxidative and endoplasmic reticulum stresses in mouse tissues: implications for the development of acute toxicity after intravenous administration. Toxicol. Res. 5 (2), 602–608. 10.1039/c5tx00464k PMC6062397 30090374

[B13] Choi H. J. Yun J. W. Kim Y. H. Kwon E. Hyon M.-K. Kim J. Y. (2019). Evaluation of acute and subacute toxicity of sodium taurodeoxycholate in rats. Drug Chem. Toxicol., 44(3), 268–276. 10.1080/01480545.2019.1609493 31215257

[B14] Dandekar P. Dhumal R. Jain R. Tiwari D. Vanage G. Patravale V. B. (2010). Toxicological evaluation of pH-sensitive nanoparticles of curcumin: acute, sub-acute and genotoxicity studies. Food Chem. Toxicol. 48 (8–9), 2073–2089. 10.1016/j.fct.2010.05.008 20470854

[B15] de Barros A. L. Cavalheiro G. F. de Souza A. V. M. Traesel G. K. Anselmo-Franci J. A. Kassuya C. A. L. (2016). Subacute toxicity assessment of diflubenzuron, an insect growth regulator, in adult male rats. Environ. Toxicol. 31 (4), 407–414. 10.1002/tox.22054 25266294

[B16] DeMattos R. B. Bales K. R. Cummins D. J. Paul S. Holtzman D. M., (2002). Brain to plasma amyloid-beta efflux: a measure of brain amyloid burden in a mouse model of alzheimer’s disease. Science 295 (5563), 2264–2267. 10.1126/science.1067568 11910111

[B17] Desai N. C. Shihora P. N. Rajpara K. M. Joshi V. V. Vaghani H. V. Satodiya H. M. (2012). Synthesis, characterization, and antimicrobial evaluation of novel naphthalene-based 1,2,4-triazoles. Med. Chem. Res. 21 (10), 2981–2989. 10.1007/s00044-011-9833-8

[B18] Ehlers K. Elmazar M. M. A. Nau H. (1996). Methionine reduces the valproic acid-induced spina bifida rate in mice without altering valproic acid kinetics. J. Nutr. 126 (1), 67–75. 10.1093/jn/126.1.67 8558327

[B19] Fu X. Ji R. Dam J. (2009). Acute, subacute toxicity and genotoxic effect of Bio-Quinone Q10 in mice and rats. Regul. Toxicol. Pharmacol. 53 (1), 1–5. 10.1016/j.yrtph.2008.09.003 18929610

[B20] Gelbke H.-P. Hofmann A. Owens J. W. Freyberger A. (2007). The enhancement of the subacute repeat dose toxicity test OECD TG 407 for the detection of endocrine active chemicals: comparison with toxicity tests of longer duration. Arch. Toxicol. 81 (4), 227–250. 10.1007/s00204-006-0148-3 17047927

[B21] OECD (2002). Test No. 423: Acute Oral Toxicity-Acute Toxic Class Method, OECD Guidelines for the Testing of Chemicals, Paris, France: OECD Publishing.

[B22] Harvey A. L. (2008). Natural products in drug discovery. Drug Discov. Today 13 (19-20), 894–901. 10.1016/j.drudis.2008.07.004 18691670

[B23] Hira S. Saleem U. Anwar F. Raza Z. Rehman A. U. Ahmad B. (2020).In Silico study and pharmacological evaluation of eplerinone as an anti-alzheimer’s drug in STZ-induced alzheimer’s disease mode. ACS Omega, 5 (23), 13973–13983. 10.1021/acsomega.0c01381 32566864 PMC7301577

[B24] Hira S. Saleem U. Anwar F. Sohail M. F. Raza Z. Ahmad B. (2019). β-Carotene: a natural compound improves cognitive impairment and oxidative stress in a mouse model of streptozotocin-induced alzheimer’s disease. Biomolecules 9 (9), 441. 10.3390/biom9090441 PMC6769610 31480727

[B25] Hughes J. Rees S. Kalindjian S. Philpott K. (2011). Principles of early drug discovery. Br. J. Pharmacol. 162 (6), 1239–1249. 10.1111/j.1476-5381.2010.01127.x 21091654 PMC3058157

[B26] Husain S. S. Pejo E. Ge R. Raines D. E. (2012). Modifying methoxycarbonyl etomidate inter-ester spacer optimizes in vitro metabolic stability and in vivo hypnotic potency and duration of action. Anesthesiology 117 (5), 1027. 10.1097/aln.0b013e31826d3bef 22929736 PMC3509384

[B27] Iman K. Mirza M. U. Mazhar N. Vanmeert M. Irshad I. Kamal M. A. (2018). In silico structure-based identification of novel acetylcholinesterase inhibitors against alzheimer’s disease. CNS Neurol. Disord. Drug Targets 17 (1), 54–68. 10.2174/1871527317666180115162422 29336270

[B28] Jones D. P. (2006). Redefining oxidative stress. Antioxid. Redox Signaling 8 (9-10), 1865–1879. 10.1089/ars.2006.8.1865 16987039

[B29] Jothy S. L. Zakaria Z. Chen Y. Lau Y. L. Latha L. Y. Sasidharan S. (2011). Acute oral toxicity of methanolic seed extract of *Cassia* fistula in mice. Molecules 16 (6), 5268–5282. 10.3390/molecules16065268 21701437 PMC6264368

[B30] Karnam S. S. Ghosh R. C. Mondal S. Mondal M. (2015). Evaluation of subacute bisphenol-a toxicity on male reproductive system. Vet. World 8 (6), 738. 10.14202/vetworld.2015.738-744 27065640 PMC4825275

[B31] Klaassen C. D. Amdur M. O. (2013). Casarett and Doull’s toxicology: the basic science of poisons. New York, NY: McGraw-Hill.

[B32] Klaassen C. (1996). Principles of toxicology and treatment of poisoning. Goodman & Gilman’s the pharmacological basis of therapeutics, New York, NY: McGraw-Hill. 361–398.

[B33] Konan N. A. Bacchi E. M. Lincopan N. Varela S. D. Varanda E. A. (2007). Acute, subacute toxicity and genotoxic effect of a hydroethanolic extract of the cashew (*Anacardium occidentale* L.). J. Ethnopharmacol. 110 (1), 30–38. 10.1016/j.jep.2006.08.033 17088034

[B34] Lewis R. W. Billington R. Debryune E. Gamer A. Lang B. Carpanini F. (2002). Recognition of adverse and nonadverse effects in toxicity studies. Toxicol. Pathol. 30 (1), 66–74. 10.1080/01926230252824725 11890477

[B35] Macklin R. (2010). Enrolling pregnant women in biomedical research. The Lancet 375 (9715), 632–633. 10.1016/s0140-6736(10)60257-7 20198725

[B36] Masic A. Hernandez A. M. V Hazra S. Glaser J. Holzgrable U. Hazra B. (2015). Cinnamic acid bornyl ester derivatives from valeriana wallichii exhibit antileishmanial *in vivo* activity in leishmania major-infected balb/c mice. PLoS One 10 (11), e0142386. 10.1371/journal.pone.0142386 26554591 PMC4640567

[B37] Menegola E. Broccia M. L. Giavini E. (2001). Atlas of rat fetal skeleton double stained for bone and cartilage. Teratology 64 (3), 125–133. 10.1002/tera.1055 11514942

[B38] Mir N. T. Saleem U. Anwar F. Ahmad B. Ullah I. Hira S. (2019). Lawsonia Inermis markedly improves cognitive functions in animal models and modulate oxidative stress markers in the brain. Medicina 55 (5), 192. 10.3390/medicina55050192 PMC6571555 31121979

[B39] Morton D. M. (1998). Importance of species selection in drug toxicity testing. Toxicol. Lett. 102–103, 545–550. 10.1016/s0378-4274(98)00263-x 10022310

[B40] Mythilypriya R. Shanthi P. Sachdanandam P. (2007). Oral acute and subacute toxicity studies with Kalpaamruthaa, a modified indigenous preparation, on rats. J. Health Sci. 53 (4), 351–358. 10.1248/jhs.53.351

[B41] Nair A. R. Lee W.-K. Smeets K. Swennen Q. Sanchez A. Thévenod F. (2015). Glutathione and mitochondria determine acute defense responses and adaptive processes in cadmium-induced oxidative stress and toxicity of the kidney. Arch. Toxicol. 89 (12), 2273–2289. 10.1007/s00204-014-1401-9 25388156

[B43] OECD Guideline for Testing of Chemicals No (1995). Repeated dose 28‐day oral toxicity Study in Rodents, 1995. Paris, France: Organisation for Economic Co-operation and Development Paris.

[B42] Rand G. M. Petrocelli S. R. (1985). Fundamentals of aquatic toxicology: methods and applications. Princeton, NJ: FMC Corp.

[B44] Saba A. B. Oridupa O Oyeyemi M O Osanyigbe O D (2009). Spermatozoa morphology and characteristics of male wistar rats administered with ethanolic extract of Lagenaria Breviflora Roberts. Afr. J. Biotechnol. 8 (7), 1170–1175.

[B45] Saeed M. Saleem U. Anwar F. Ahmad B. Anwar A. (2020). Inhibition of valproic acid-induced prenatal developmental abnormalities with antioxidants in rats. ACS omega 5 (10), 4953–4961. 10.1021/acsomega.9b03792 32201781 PMC7081441

[B46] Saleem U. Raza Z. Anwar F. Ahmad B. Hira S. Ali T. (2019). Experimental and computational studies to characterize and evaluate the therapeutic effect of albizia lebbeck (L.) seeds in alzheimer’s disease. Medicina 55 (5), 184. 10.3390/medicina55050184 PMC6572470 31117312

[B47] Shang E. Liu Y. Wu Y. Zhu W. He C. Lai L. (2014). Development of 3,5-dinitrobenzoate-based 5-lipoxygenase inhibitors. Bioorg. Med. Chem. 22 (8), 2396–2402. 10.1016/j.bmc.2014.03.008 24685113

[B48] Steinberg P. van der Voet H. Goedhart P. W. Kleter G. Kok E. J. Pla M. (2019). Lack of adverse effects in subchronic and chronic toxicity/carcinogenicity studies on the glyphosate-resistant genetically modified maize NK603 in Wistar Han RCC rats. Arch. Toxicol. 93 (4), 1095–1139. 10.1007/s00204-019-02400-1 30756133 PMC7261740

[B49] Thenmozhi A. J. Raja T. R. W. Janakiraman U. Manivasagam T. (2015). Neuroprotective effect of hesperidin on aluminium chloride induced alzheimer’s disease in wistar rats. Neurochem. Res. 40 (4), 767–776. 10.1007/s11064-015-1525-1 25630717

[B50] Ugwah-Oguejiofor C. J. Okdi C. O. Ugwah M. O. Umaru M. L. Ogbulie C. S. Umar M. (2019). Acute and sub-acute toxicity of aqueous extract of aerial parts of Caralluma dalzielii NE Brown in mice and rats. Heliyon 5 (1), e01179. 10.1016/j.heliyon.2019.e01179 30775575 PMC6356088

[B51] Variya B. C. Bakrania A. K. Madan P. Patel S. S. (2019). Acute and 28-days repeated dose sub-acute toxicity study of gallic acid in albino mice. Regul. Toxicol. Pharmacol. 101, 71–78. 10.1016/j.yrtph.2018.11.010 30465803

[B52] Yamasaki K. Sawaki M. Noda S. Imatanaka N. Takatsuki M. (2002). Subacute oral toxicity study of ethynylestradiol and bisphenol A, based on the draft protocol for the “Enhanced OECD Test Guideline no. 407”. Arch. Toxicol. 76 (2), 65–74. 10.1007/s00204-001-0319-1 11914775

[B53] Zhang C. Zhang J. Zhu L. Du Z. Wang J. Wang J. (2020). Fluoxastrobin-induced effects on acute toxicity, development toxicity, oxidative stress, and DNA damage in *Danio rerio* embryos. Sci. Total Environ. 715, 137069. 10.1016/j.scitotenv.2020.137069 32041080

